# Negative pressure pulmonary oedema

**DOI:** 10.4103/0019-5049.76566

**Published:** 2011

**Authors:** Mukul C Kapoor

**Affiliations:** Department of Anaesthesiology, Command Hospital (CC), Lucknow, Uttar Pradesh, India. E-mail: mukulanjali@rediffmail.com

Pulmonary oedema is defined as abnormal accumulation of fluid in the extravascular compartment of the lung. Negative pressure pulmonary oedema (NPPO) represents a pure form of hydrostatic oedema.[1] NPPO has also been referred to as ‘post-obstructive pulmonary oedema’ and ‘laryngospasm-induced pulmonary oedema’. It characteristically occurs after relief of upper airway obstruction, generally from laryngospasm. Other reported causes of NPPO are impacted foreign body trachea/bronchus, epiglottitis, strangulation, vocal cord palsy, acromegaly, goitre, inspissated tracheal secretions, direct suctioning of the endotracheal tube adapter, hiccoughs, croup, temporomandibular joint arthroscopy, difficult intubation, haematoma, upper airway tumour, oropharyngeal surgery, Ludwig angina, obesity, obstructive sleep apnoea, mediastinal tumour, biting/obstruction of the endotracheal tube/laryngeal mask and falling back of tongue.[1,2]

The incidence of NPPO, as a complication of all anaesthetics, is said to be 0.05-0.1%.[2] It has however been suggested that the occurrence of NPPO is under-reported, as it is often unrecognized or misdiagnosed.[2] The morbidity and mortality of an un-recognized event of NPPO is as high as 40%.[3] Patients are usually young, athletic males capable of generating profound negative intra-thoracic pressure (ITP).[4] (Healthy human subjects can generate negative ITP to a reported maximum of -140 cm H_2_O.)[5]

In case the obstruction is primarily during inspiration, the patient tries to overcome the obstruction with a forced inhalation effort (Muller manoeuvre). This results in development of high negative ITP and a rise in venous return, which induces hydrostatic transudation of fluid into the extra-vascular compartment. In case there is obstruction during both inspiration and expiration, high positive ITP prevents the development of oedema. However, oedema develops on relief of the obstruction due to a sudden fall in the ITP.[6] It has been suggested that the aetiology of pulmonary oedema represents an interplay between several factors, which include cardiogenic and neurogenic mechanisms, as well as hypoxia.[7]

Although negative ITP is the primary pathological event in the genesis of NPPO, hypoxia, hypercarbia, acidosis, and hyperadrenergic state contribute to its development. Increased right heart filling, decreased left heart filling, increased left ventricular afterload and decreased LV ejection lead to increased pulmonary capillary hydrostatic pressure.[8] Hypoxaemia increases pulmonary vascular resistance resulting in a rise in pulmonary capillary wedge pressure. Hypoxaemia alters the capillary integrity and precipitates a hyperadrenergic status. This along with the hypercarbia, redistributes blood from the systemic circulation to the pulmonary circulation. Hypoxaemia and acidaemia have myocardial depressant effects, which also contributes to pulmonary oedema formation.[9,10] Another potential cause cited is capillary leak due to capillary disruption from shear stretching forces of high ITP or extreme hypoxia.[11] However, a recent study has established that hydrostatic forces are the primary mechanism behind NPPO and that the alveolar epithelium remains functionally intact in acute NPPO.[1] The combination of increased preload and afterload leads to a marked rise in hydrostatic pressure in the pulmonary microvasculature, and, as dictated by the Starling equation, fluid filters out of the microcirculation into the lung interstitium.[1]

The most common clinical presentation of NPPO is the occurrence of airway obstruction on emergence from general anaesthesia, followed by the rapid onset of respiratory distress, haemoptysis and clinical/radiological features consistent with bilateral pulmonary oedema. Typically, NPPO radiologically manifests as Kerley lines, peribronchial cuffing and, in severe cases, as central alveolar oedema. Cardiac size is usually normal as hypervolaemia is not present.[6] Resolution of the clinical and radiological features is rapid, usually within 24 h.

Radiologically, the two patho-physiological phases in the development of pressure oedema are interstitial oedema and alveolar flooding. Interstitial oedema occurs with a rise in transmural arterial pressure of 15-25 mmHg and is characterized by the appearance of peribronchial cuffing, Kerley lines and subpleural effusion. With increases in transmural pressure to greater than 25 mmHg, there is extension of oedema into alveolar spaces, creating nodular areas of opacity that coalesce into frank consolidations.[6]

Differential diagnoses of NPPO include aspiration pneumonitis, acute lung injury, cardiogenic pulmonary oedema, fluid overload, drug-induced non-cardiogenic pulmonary oedema and anaphylaxis. Treatment modalities range from oxygen supplementation through mask, to mask CPAP, tracheal intubation and positive pressure ventilation. Some cases require minimal supportive care, including maintenance of a patent airway and administration of supplemental oxygen; however, most of the patients require reintubation and ventilation with positive airway pressure.[4] It is important to apply positive pressure to the airway early. Nasal bi-level positive airway pressure, mask CPAP and intubation and ventilation with PEEP have been used successfully.[2] Diuretics are often administered but their role is uncertain.[4] Early relief of laryngospasm with neuromuscular blockers has been suggested to “break” the laryngospasm and put a stop to the sustained negative ITP, hyperadrenergic drive and hypoxia implicated in the pathogenesis of NPPO.[2]
